# Use of Exopolysaccharide-Synthesizing Lactic Acid Bacteria and Fat Replacers for Manufacturing Reduced-Fat Burrata Cheese: Microbiological Aspects and Sensory Evaluation

**DOI:** 10.3390/microorganisms8101618

**Published:** 2020-10-21

**Authors:** Giuseppe Costantino, Maria Calasso, Fabio Minervini, Maria De Angelis

**Affiliations:** Department of Soil, Plant and Food Sciences, University of Bari Aldo Moro, Via Amendola 165/a, 70126 Bari, Italy; giuseppecostant1989@libero.it (G.C.); maria.calasso@uniba.it (M.C.); maria.deangelis@uniba.it (M.D.A.)

**Keywords:** reduced-fat cheese, Burrata cheese, fat replacers, exopolysaccharides-synthesizing lactic acid bacteria, characterization of microbiota, sensory analyses

## Abstract

This study aimed to set-up a biotechnological protocol for manufacturing a reduced-fat Burrata cheese using semi-skimmed milk and reduced-fat cream, in different combinations with exopolysaccharides-synthesizing bacterial starters (*Streptococcus thermophilus*, E1, or *Lactococcus lactis* subsp. *lactis* and *Lc*. *lactis* subsp. *cremoris*, E2) and carrageenan or xanthan. Eight variants of reduced-fat cheese (fat concentration 34–51% lower than traditional full-fat Burrata cheese, used as the control) were obtained using: (i) semi-skimmed milk and reduced-fat cream alone (RC) or in combination with (ii) xanthan (RCX), (iii) carrageenan (RCC), (iv) starter E1 (RCE1), (v) starter E2 (RCE2), (vi) both starters (RCE1-2), (vii) E1 and xanthan (RCXE1), or E1 and carrageenan (RCCE1). Post-acidification occurred for the RCC, RCX, and RCE2 Burrata cheeses, due to the higher number of mesophilic cocci found in these cheeses after 16 days of storage. Overall, mesophilic and thermophilic cocci, although showing cheese variant-depending dynamics, were dominant microbial groups, flanked by *Pseudomonas* sp. during storage. Lactobacilli, increasing during storage, represented another dominant microbial group. The panel test gave highest scores to RCE1-2 and RCXE1 cheeses, even after 16 days of storage. The 16S-targeted metagenomic analysis revealed that a core microbiota (*S. thermophilus*, *Streptococcus lutetiensis*, *Lc. lactis*, *Lactococcus* sp., *Leuconostoc lactis*, *Lactobacillus delbrueckii*, and *Pseudomonas* sp.), characterized the Burrata cheeses. A consumer test, based on 105 people, showed that more than 50% of consumers did not distinguish the traditional full-fat from the RCXE1 reduced-fat Burrata cheese.

## 1. Introduction

Burrata is a fresh “pasta filata” (i.e., stretched curd) cheese that originated in Apulia region at the beginning of nineteenth century [1]. To date, it is considered a specialty of Southern Italy, being produced, also, in Campania and Basilicata. Burrata cheese is included in the list of traditional agri-food products (Prodotto Agroalimentare Tradizionale (PAT) [2]. Moreover, a variety of Burrata, “Burrata di Andria”, received the Protected Geographical Indication [3]. Burrata cheese is manufactured using pasteurized cow’s milk, which is usually mixed with the acidified whey milk of the previous day’s cheese-making process and added with calf rennet [4]. Once curd is obtained, a treatment with hot (80–90 °C) water allows stretching the curd. Then, the cheese is molded in its characteristic double structure, which consists of a “bag”, made of stretched curd, and an inner creamy core, consisting of a mixture of strips of stretched curd (“sfilacci”) and milk cream. Finally, the cheese is cooled down in chilled water [5].

Dairy products, including cheeses, provide nutrients to humans [6]. They are rich in proteins, lipids, many minerals (including Ca, Mg, P, and Zn) and vitamins (e.g., A, D, E, and K) [7]. Cheeses represent the main source of dietary Ca in many countries, especially USA, UK and Northern Europe [8,9]. Despite the important nutritional value of cheese, some researchers observed that its consumption increased in developing countries, whereas it showed a slight decline in developed countries [10]. This trend may be linked to the fact that a large dietary consumption of cheeses may play a negative role on health [11], because of the fat content, reaching high values (60% of dry matter) in some varieties, such as Burrata [1]. Worldwide prevalence of obesity has been associated to the unbalance of dietary patterns (preference for high fat and sugar content food) and lifestyles [12]. Moreover, the combination of an unhealthy diet and factors, such as genetic background, smoking, and environmental pollution, leads to heart diseases and several types of cancers [13]. In particular, dietary fat has been linked with various breast, colorectal, pancreatic and prostate malignant cancers [14,15,16,17]. In addition, consumption of high fat and/or sugar induces a low-grade intestinal inflammation in animal model and leads to a change in the gut microbiota [12,18,19,20]. Furthermore, both a diet high in fat and obesity may have detrimental effects on sperm quality, resulting in a reduced mating and fertilization success [21]. Last, but not least, large intake of fat is directly linked to cardiovascular disease [22]. To date, the reduction of total dietary fat to less than 30% of total energy is recommended worldwide [23], including in countries where the Mediterranean diet is usually adopted [24].

Although numerous studies focused on strategies to reduce fat in cheese [25,26,27,28], commercial low-fat cheeses are perceived by consumers as excessively dry, firm, or difficult to chew, and with atypical flavor [26,27,28,29]. Indeed, fat positively affects the body, texture (e.g., palatability) and rheological properties of cheeses [30], by filling the interstitial spaces of casein network during curd coagulation, thus preventing the caseins from clumping too much [31,32,33,34]. Fat-replacers (e.g., carbohydrates, protein-, or fat-based compounds) increase the moisture content of low-fat cheeses and mimic the role of fat on sensory properties [27,35,36,37,38]. For instance, microparticulated whey proteins and starters producing exopolysaccharides (EPS) were used as fat replacers in Caciotta cheeses [39]. Trani et al. [1] suggested the use of a mixture of carob flour suspension and milk cream as ingredient of the inner core of Burrata cheeses. However, the resulting cheese, although acceptable, was perceived different from the full-fat cheese [1].

The aim of this work was to set-up a biotechnological protocol for manufacturing a reduced-fat Burrata cheese, sensorially indistinguishable from its traditional, full-fat, counterpart, with minimal modifications of the standard cheese-making processing. To this aim, different combinations of EPS-synthesizing lactic acid bacteria and natural fibers were tested as fat-replacers.

## 2. Materials and Methods

### 2.1. Microorganisms

Commercially available deep-frozen exopolysaccharide (EPS) producing starters, Cryofast ST440 and Cryofast MO342, were purchased from Clerici, Sacco S.r.l. (Cadorago, Como, Italy): Cryofast ST440 (alias E1) contains *Streptococcus thermophilus*, and Cryofast MO342 (alias E2) contains *Lactococcus lactis* subsp. *lactis* and *Lc. lactis* subsp. *cremoris*.

### 2.2. Manufacturing of Burrata Cheese

Whole milk (lactose 4.90%, protein 3.70%, fat 4.46%, pH 6.6, and cell density of total aerophilic microorganisms of ca. 4.3 log CFU g^−1^), supplied by Masseria Foggia Nuova (Noci, Bari, Italy), was treated through a Westfalia Separator MSB60-01-076 (GEA Group, Oelde, Germany) to obtain skimmed milk. Before being used, whole milk and skimmed milk were mixed in a ratio of 50:50 in order to obtain a semi-skimmed milk (ca 2.23% fat content). Subsequently, whole milk and semi-skimmed milk were pasteurized (72 °C for 15 s) using a plate heat exchanger and then instantaneously cooled at 37–38 °C. Milk was either directly acidified with lactic acid, reaching value of pH of ca. 5.90, or biologically acidified to pH of ca. 5.70 upon addition of E1 commercial starter. Three batches of milk were used for manufacturing as many batches of Burrata cheeses. No significant differences (*p* > 0.05) were observed among the three batches of milk, in terms of gross composition, pH, and cell density of total aerophilic microorganisms.

Two types of powdered fat-replacers were used during manufacturing of four out of nine Burrata cheeses: (i) xanthan gum powder (99% fiber purity) and (ii) carrageenan gum powder (99% fiber purity), both purchased from Farmalabor S.r.l. (Canosa di Puglia, Barletta-Andria-Trani, Italy). Four types of milk cream were used for filling the inner core of Burrata cheese: (conventional) cream (28% fat content), purchased from Cerri S.r.l. (Buronzo, Vercelli, Italy); reduced-fat cream (22% fat content) (Cerri S.r.l.); reduced-fat cream diluted (60:40) with xanthan (0.5% *wt*/*vol*) suspension (14% fat content); reduced-fat cream diluted (60:40) with carrageenan (1.0% *wt*/*vol*) suspension (14% fat content).

Nine types (including a control) of experimental Burrata cheeses were manufactured at the industrial plant Ignalat, located in Noci (Bari, Italy) (Table S1), namely: (1) cheese whose curd and “sfilacci” were obtained from whole milk, and whose core was constituted of “sfilacci” and cream (control); (2) cheese whose curd and “sfilacci” were obtained from semi-skimmed milk, and whose core was constituted of “sfilacci” and reduced-fat cream (RC); (3) cheese whose curd and “sfilacci” were obtained from semi-skimmed milk, and whose core was constituted of “sfilacci” and reduced-fat cream and xanthan (RCX); (4) cheese whose curd and “sfilacci” were obtained from semi-skimmed milk, and whose core was constituted of “sfilacci” and reduced-fat cream and carrageenan (RCC); (5) cheese whose curd and “sfilacci” were obtained from semi-skimmed milk inoculated with the starter E1 (6% *wt/vol*), and whose core was constituted of “sfilacci” and reduced-fat cream (RCE1); (6) cheese whose curd and “sfilacci” were obtained from semi-skimmed milk, and whose core was constituted of “sfilacci” and reduced-fat cream inoculated with the starter E2 (3% *wt*/*vol*) (RCE2); (7) cheese whose curd and “sfilacci” were obtained from semi-skimmed milk inoculated with the starter E1 (6% *wt*/*vol*), and whose core was constituted of “sfilacci” and reduced-fat cream inoculated with the starter E2 (3% *wt*/*vol*) (RCE1-2); (8) cheese whose curd and “sfilacci” were obtained from semi-skimmed milk inoculated with the starter E1 (6% *wt*/*vol*), and whose core was constituted of “sfilacci” and reduced-fat cream and xanthan (RCXE1); (9) cheese whose curd and “sfilacci” were obtained from semi-skimmed milk inoculated with the starter E1 (6% *wt*/*vol*), and whose core was constituted of “sfilacci” and reduced-fat cream and carrageenan (RCCE1). After manufacturing, all the cheeses were stored at 4 °C for 16 days.

### 2.3. Compositional Analysis

The Burrata cheeses were analyzed for the concentration of total carbohydrates, through high-pressure liquid chromatography, and proteins, through Kjeldahl method, as described by Baldini et al. [40]. Fat content was determined using the van Gulik method [41]. Moisture was determined using Moisture Analyzer MA35 (Sartorius Stedim Biotech GmbH, Germany). The pH value was measured by direct insertion of a FoodTrode Electrode (Hamilton, Bonaduz, Switzerland). Total titratable acidity (TTA) was determined on 10 g of sample, after homogenization with 90 mL of distilled water and expressed as the amount (mL) of 0.1 M NaOH necessary to get pH of 8.3.

### 2.4. Assessment of Primary Proteolysis

The pH 4.6-soluble and -insoluble nitrogen fractions of cheeses, obtained according to the method described by Kuchroo and Fox [42], were analyzed by denaturing urea polyacrylamide gel electrophoresis (urea-PAGE) [43], using sodium caseinate as standard reference. The gels were stained using Coomassie Brilliant blue G250 colorant, and destained according to Blakesley and Boezi [44]. Protein content of the pH 4.6-soluble and -insoluble nitrogen fractions of cheeses was evaluated spectrophotometrically by the Bradford method [45]. Concentration of peptides in the pH 4.6-soluble fraction was estimated by *o*-phthalaldehyde spectrophotometer method [46].

### 2.5. Cultivable Microbiota

Microbiological analyses were carried out as previously described by Minervini et al. [47] using culture media and supplements purchased from Oxoid (Basingstoke, UK). Ten grams of Burrata cheese were homogenized with 90 mL of sterile saline (NaCl, 9 g L^−1^) in a 400P Bag Mixer (3 min of treatment). Cell density of total mesophilic aerobic microorganisms was determined using plate count agar after incubation at 30 °C. Presumptive mesophilic and thermophilic lactobacilli were enumerated using de Man, Rogosa and Sharpe (MRS) agar plates with the addition of cycloheximide (0.1% *wt*/*vol*) and incubated at 30 °C and 45 °C, respectively. Presumptive mesophilic and thermophilic cocci were enumerated using lactose M17 agar plates with the addition of cycloheximide (0.1% *wt*/*vol*) and incubated at the same temperatures as for presumptive lactobacilli. Enterococci were counted after inoculating, by spreading technique, plates of Slanetz and Bartley agar and incubating at 37 °C. Staphylococci were determined inoculating, by spreading, plates of Baird Parker agar supplemented with egg yolk tellurite, and incubating at 37 °C. Total coliforms were counted on violet red bile glucose agar (VRBGA) after incubating plates at 37 °C. Plates of *Pseudomonas* agar, supplemented with cetrimide, fucidin, and cephalosporin (CFC) supplement, were spread inoculated with 0.1 mL of diluted sample and used to enumerate *Pseudomonas* spp. after incubation at 30 °C. Yeasts were enumerated on Sabouraud Dextrose Agar plates with the addition of chloramphenicol (0.1% *wt*/*vol*) and incubated at 25 °C. All plates were incubated for 48 h, except for VRBGA and *Pseudomonas* agar, which were incubated for 24 h.

### 2.6. Sensory Analysis

The sensory analysis of Burrata cheese was carried out using the method described by Coppola et al. [48] as modified by De Angelis et al. [49]. Ten volunteers (5 males and 5 females), with mean age of 30 years (range: 20–40 years), were recruited from the laboratory staff. Three introductory sensory training sessions were held for discussing the sensory attributes with the panelists. Cheeses were taken out of the refrigerator 1 h before the sensory evaluation, and served at room temperature under normal (daylight) illumination. Each cheese (two pieces per thesis), identified by a code number, was given to each panelist on a single tray. Samples were served in a random order and evaluated in two replicates by all panelists. The quality attributes evaluated were governing liquid transparency, color, surface appearance, elasticity, sliminess, cream milk odor, fermented milk odor, acid taste, bitter taste, sweet taste, cream milk taste, salty taste, and aftertaste. Each sensory trait was rated with a score from 1 (lowest) to 5 (highest).

An additional sensory analysis, namely a consumer test, was implemented regarding the control and RCXE1 Burrata cheeses stored for 2 days at 4 °C. One hundred and five people, who regularly consume fresh cheeses, were voluntarily recruited. All of the consumers tasted both the traditional full-fat (control) and the RCXE1 (reduced-fat) Burrata cheeses. Both the Burrata cheeses were labelled under different non-allusive codes, respectively “A” for control and “B” for RCXE1. Before the test, consumers were asked to fill in an evaluation form, which included several questions, such as age, educational qualification, job type, degree of knowledge of Burrata cheese, and purchasing and consumption habits. The sensory evaluation was conducted in a conference room where temporary partitions were erected to create one-consumer tasting booths, in order to reduce misperception and avoid crossed bias [50]. Each consumer was asked to distinguish, at first sight and taste, the traditional full-fat Burrata cheese from the reduced-fat one. Afterwards, the control and reduced-fat cheese were singly disclosed to each recruited consumer and consumer was asked to express compared judgment about appearance, texture, and odor, using one of the following phrases: (i) preference for control (Control); (ii) preference for reduced-fat cheese (RCXE1); (iii) no difference between the two cheeses (indifferent). Finally, consumers expressed the overall acceptability on a 0–10 point hedonic scale, taking into account appearance, texture, odor, and taste of Burrata cheeses.

### 2.7. Extraction and Sequencing of Total Bacteria DNA

Total DNA was extracted from Burrata cheese samples using FastDNA Spin Kit (MP Biomedicals, Solon, OH, USA) according to the manufacturer instructions. Quality and concentration of DNA was evaluated spectrophotometrically (NanoDrop ND-1000, Thermo Fisher Scientific, Inc., Waltham, MA, USA). DNA was used as template for 16S metagenomic analysis, which was carried out by Research and Testing Laboratory (RTL, Lubbock, TX, USA), using the Illumina MiSeq platform. A fragment of the 16S DNA gene for analysis of the diversity inside the domain of *Bacteria* was amplified using the primers 28F (GAGTTTGATCNTGGCTCAG) [51] and 519R (GTNTTACNGCGGCKGCTG) [52]. PCR and sequencing analyses were carried out according to the protocol of RTL. The sequenced reads were subsequently merged by PEAR Illumina paired-end merger and subsequently subjected to the USEARCH algorithm [53], which groups reads into clusters that include reads showing not more than 4% dissimilarity. Operational Taxonomic Units (OTUs) were selected by using the UPARSE OTU selection algorithm and the selected OTUs were chimera-checked using the UCHIME software, executed in *de novo* mode [54]. The percentage of each bacterial OTU was analyzed individually for each sample, providing relative abundance information among the samples, based on the relative numbers of reads within each [55].

### 2.8. Statistical Analyses

Data (at least three biological replicates) were subjected to one-way analysis of variance (ANOVA), and pair comparison of treatment means was achieved by Tukey’s procedure at *p* < 0.05, using the statistical software Statistica v. 7.0 for Windows. Principal Component Analysis (PCA) was also performed using Statistica v. 7.0. Spearman correlations for cell densities of microbial groups, OTUs, and biochemical characteristics of Burrata cheeses were computed using Statistica v. 7.0 and elaborated through PermutMatrix software.

## 3. Results

### 3.1. Compositional Analysis

After 1 day of manufacturing, all nine Burrata cheeses shared average values of total carbohydrates and proteins of 1.06 ± 0.6 g/100 g and 10.77 ± 0.3 g/100 g, respectively. Control cheese (represented by traditional full-fat Burrata cheese) contained ca. 22% of fat (Table 1), whereas the Burrata cheeses obtained from semi-skimmed milk and reduced-fat cream, without adding xanthan or carrageenan in cheese core (RC, RCE1, RCE2, RCE1-2), contained on average 14.5% of fat, corresponding to ca. 34% in fat reduction. Burrata cheeses obtained from semi-skimmed milk and reduced-fat cream and with addition of xanthan or carrageenan (RCX, RCC, RCXE1, and RCCE1) showed an average fat concentration of 10.47%, corresponding to ca. 51% in fat reduction. Moisture ranged between ca. 65% (control Burrata cheese) to ca. 75% (RCXE1) (Table 1).

**Figure 1:**
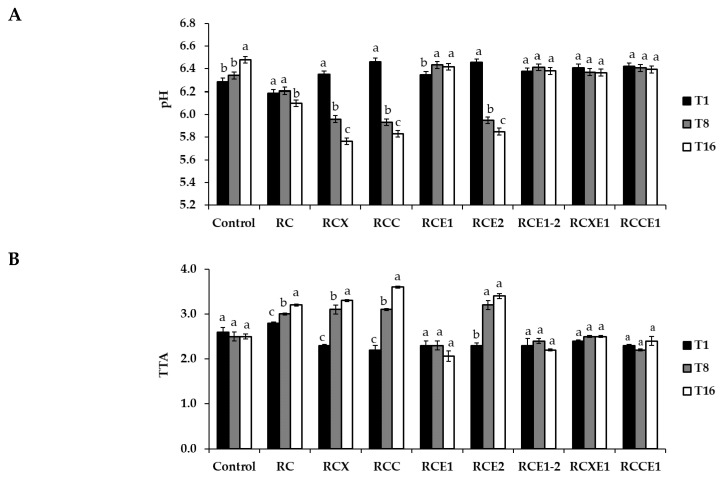
Values of pH (**A**) and total titratable acidity (TTA) (**B**), determined after 1 (T1), 8 (T8), and 16 (T16) days of storage at 4 °C, in the Burrata cheeses made from whole milk and cream (Control); semi-skimmed milk and reduced-fat cream (RC); semi-skimmed milk and reduced-fat cream diluted with xanthan (RCX) or carrageenan (RCC); semi-skimmed milk added with exopolysaccharide producing starter E1 and reduced-fat cream (RCE1); semi-skimmed milk and reduced-fat cream added with exopolysaccharide producing starter E2 (RCE2); semi-skimmed milk and reduced-fat cream both added with E1 and E2 (RCE1-2); semi-skimmed milk added with E1 and reduced-fat cream diluted with xanthan (RCXE1) or carrageenan (RCCE1). Within the same thesis, bars labelled with the same letter represent not significantly (*p* > 0.05) different values.

After 1 day of manufacturing, pH values ranged between 6.19 ± 0.03 (RC) and 6.46 ± 0.03 (RCC) (Figure 1A). After 8 and 16 days of storage, no statistically significant difference (*p* > 0.05) was found for RCE1-2, RCXE1, and RCCE1 cheeses. The pH of control Burrata cheese increased (*p* < 0.05) after 16 days of storage. The pH of RCE1 increased (*p* < 0.05) after 8 days, but showed no further variation (*p* > 0.05) after 16 days. With respect to day 1, a significant decrease of pH was found after 16 days for RCX (5.76 ± 0.03), RCC (5.83 ± 0.03) and RCE2 (5.85 ± 0.03) Burrata cheeses.

Total Titratable Acidity (TTA) ranged between 2.20 ± 0.10 and 2.80 ± 0.10 mL of NaOH 0.1 M, after 1 day of storage. During storage, TTA increased (*p* < 0.05) in RC, RCX, RCC, and RCE2 Burrata cheeses, especially at 8 days. Overall, no differences (*p* > 0.05) were found in the other cheeses during storage (Figure 1B).

### 3.2. Assessment of Proteolysis

**Figure 2:**
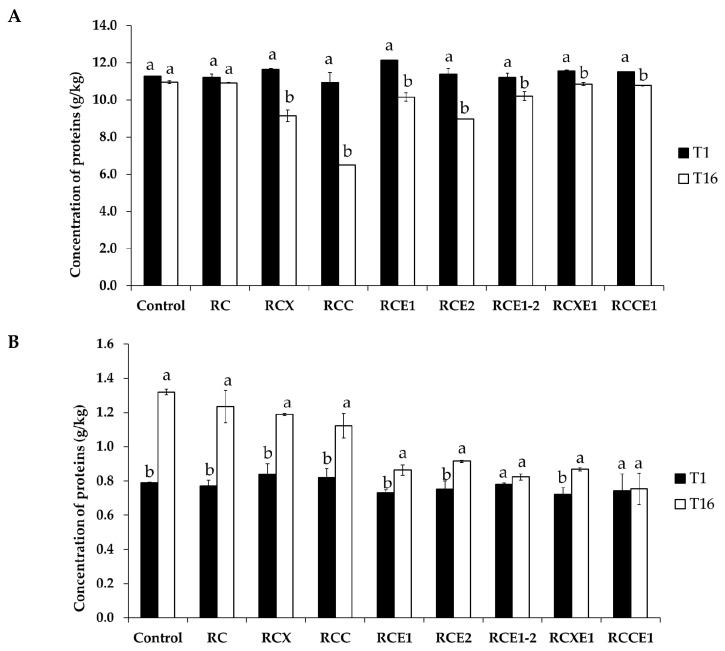
Concentration of proteins in the pH 4.6-insoluble (**A**) and pH 4.6-soluble nitrogen fraction (**B**), determined after 1 (T1) and 16 (T16) days of storage at 4 °C, in the Burrata cheeses made from whole milk and cream (Control); semi-skimmed milk and reduced-fat cream (RC); semi-skimmed milk and reduced-fat cream diluted with xanthan (RCX) or carrageenan (RCC); semi-skimmed milk added with exopolysaccharide producing starter E1 and reduced-fat cream (RCE1); semi-skimmed milk and reduced-fat cream added with exopolysaccharide producing starter E2 (RCE2); semi-skimmed milk and reduced-fat cream both added with E1 and E2 (RCE1-2); semi-skimmed milk added with E1 and reduced-fat cream diluted with xanthan (RCXE1) or carrageenan (RCCE1). Within the same thesis, bars labelled with the same letter represent not significantly (*p* > 0.05) different values.

Limited hydrolysis of casein after 1 and 16 days of storage was found for pH 4.6-insoluble nitrogen fractions extracted from the Burrata cheeses, as assessed through urea polyacrylamide gel electrophoresis (urea-PAGE) (data not shown). At day 1, pH 4.6-insoluble fraction had a protein content higher than 11 g/kg in all of the Burrata cheese variants, with slight differences among them (Figure 2A). After 16 days, the protein content generally decreased, with significant (*p* < 0.05) differences for all of the Burrata cheese variants, except for control and RC. At day 1, the concentration of proteins in the pH 4.6-soluble N fraction was ever higher than 0.7 g/kg (Figure 2B). After 16 days, overall, the protein content increased, with significant (*p* < 0.05) differences for all of the Burrata cheese variants, except for RCE1-2 and RCCE1. Overall, the cheese variants obtained using E1 and/or E2 bacterial starters showed lower protein concentration (average value: 0.85 g/kg) in the soluble fraction than the other theses (average value: 1.22 g/kg). At 1 day, peptide concentration in the soluble fraction was <300 mg/kg in RCX, RCC, whereas in the other Burrata cheese variants it ranged from ca. 310 (RCXE1) to ca. 450 (RCE1-2) mg/kg (Figure S1). After 16 days, peptides ranged from ca. 400 (RCC) to ca. 570 (RCE2) mg/kg. Except for the control, RCE1 and RCE1-2 Burrata cheeses, peptide concentration was higher (*p* < 0.05) than those found at 1 day.

### 3.3. Cultivable Microbiota

**Figure 3:**
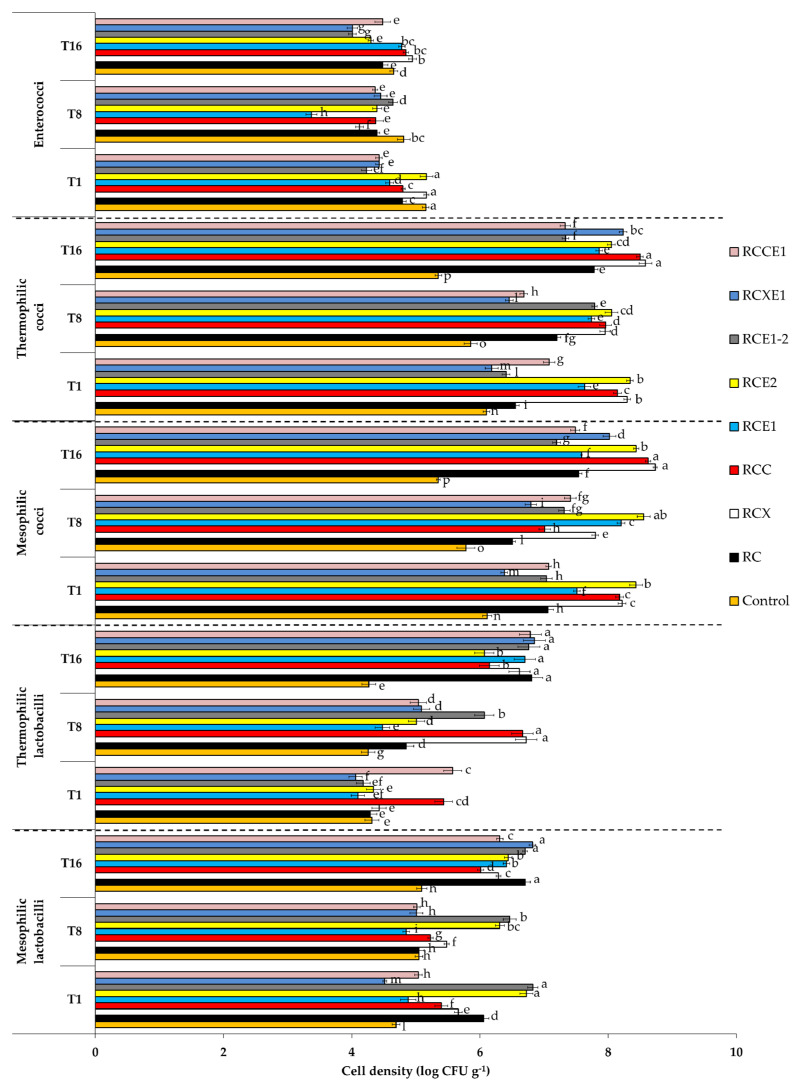

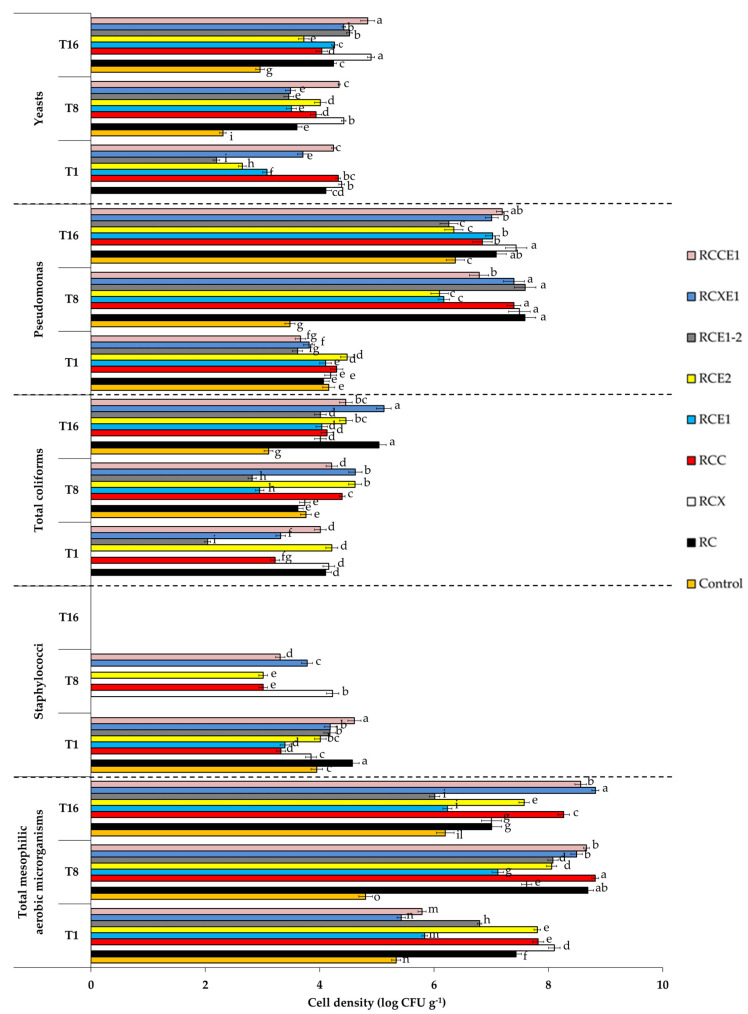
Cell densities of microbial groups, determined after 1 (T1), 8 (T8), and 16 (T16) days of storage at 4 °C, in the Burrata cheeses made from whole milk and cream (Control); semi-skimmed milk and reduced-fat cream (RC); semi-skimmed milk and reduced-fat cream diluted with xanthan (RCX) or carrageenan (RCC); semi-skimmed milk added with exopolysaccharide producing starter E1 and reduced-fat cream (RCE1); semi-skimmed milk and reduced-fat cream added with exopolysaccharide producing starter E2 (RCE2); semi-skimmed milk and reduced-fat cream both added with E1 and E2 (RCE1-2); semi-skimmed milk added with E1 and reduced-fat cream diluted with xanthan (RCXE1) or carrageenan (RCCE1). Within the same panel (showing a given microbial group), bars labelled with one or more common letters represent not significantly (*p* > 0.05) different values.

Culture-dependent microbiological analyses were carried out on all of the Burrata cheeses, after 1, 8, and 16 days from manufacturing. Cell density of presumptive mesophilic lactobacilli ranged from 4.5 log CFU g^−1^ (RCXE1) to ca. 6.8 log CFU g^−1^ (RCE1-2 Burrata cheese) after 1 day from manufacturing (Figure 3). During storage at 4 °C, this bacterial group trended to increase, especially in RCE1 and RCXE1. The only exceptions were found for RCE1-2 and RCE2, which showed no variation (*p* > 0.05) or a decrease (*p* < 0.05), respectively, of the number of mesophilic lactobacilli. At 1 day, presumptive thermophilic lactobacilli varied from 4.0 (RCXE1) to 5.6 (RCCE1) log CFU g^−1^. During storage, they trended to increase in all of the Burrata cheeses, except for the control. After 16 days, this bacterial group ranged between 4.2 (control) and 6.8 (RC, RCE1-2, RCCE1, and RCXE1) log CFU g^−1^. At 1 day, presumptive mesophilic cocci varied from 6.1 (control) to 8.4 (RCE2) log CFU g^−1^. During storage, they showed different trends depending on the Burrata cheese variant: overall decrease (control); decrease at 8 days, followed by increase at 16 days (RC, RCX, RCC); no significant (*p* > 0.05) variations (RCE1, RCE2); overall increase (RCE1-2, RCXE1, RCCE1). At 1 day, presumptive thermophilic cocci ranged between 6.1 (control) and 8.3 log CFU g^−1^ (RCX and RCE2). During storage, their trend was variable depending on the Burrata cheese variant: overall decrease (control and RCE2); decrease at 8 days, followed by increase at 16 days (RCX, RCC, RCCE1); increase at 8 days, followed by decrease at 16 days (RCE1-2); no significant (*p* > 0.05) variations (RCE1); steady increase (RC, RCXE1) (Figure 3).

Presumptive enterococci were found at relatively low cell densities in all of the Burrata cheeses and showed little variations during storage. On average, values were ca. 4.7, 4.3 and 4.5 log CFU g^−1^, after 1, 8 and 16 days, respectively. After 1 day of manufacturing, total mesophilic microorganisms ranged between 5.3 log CFU g^−1^ (control) and 8.1 log CFU g^−1^ (RCX). They trended to increase in control, RCC, RCE1, RCXE1, and RCCE1, whereas they trended to decrease in RC, RCX, and RCE1-2 Burrata cheeses. At 1 day, presumptive staphylococci were detected at cell densities ranging from 3.3 (RCC) to 4.6 (RC and RCCE1) log CFU g^−1^. After 8 days, they were not detectable in RC, RCE1, RCE1-2, and control Burrata cheeses, but persisted at an average value of ca 3.5 log CFU g^−1^ in the other cheese variants. Staphylococci were absent after 16 days in all of the cheeses. Total coliforms were not found in control and RCE1 cheeses at 1 day of manufacturing and ranged between 2.0 (RCE1-2) to 4.2 (RCE2) log CFU g^−1^ for the other Burrata cheeses. They overall increased (*p* < 0.05) during storage at 4 °C, with final values ranging between 3.1 (control) and 5.1 (RCXE1) log CFU g^−1^. The only exception was RCX, wherein coliforms showed no significant (*p* > 0.05) variation. Presumptive *Pseudomonas* sp. was found at average cell density of 4.0 log CFU g^−1^ at 1 day of storage time. During storage, overall, they increased and were detected at cell densities between ca. 6.3 (RCE1-2) and 7.4 (RCX) log CFU g^−1^ (Figure 3).

No molds were detectable after 1, 8, and 16 days of storage in all of the Burrata cheese variants (data not shown). Yeasts were not found in the control at 1 day of manufacturing, whereas in the other variants they ranged from 2.2 (RCE1-2) to 4.4 (RCX) log CFU g^−1^. During storage, yeasts increased in all of the Burrata cheese variants, with the exceptions of RC and RCC, wherein they remained constant or decreased, respectively (Figure 3).

### 3.4. Panel Test

**Figure 4:**
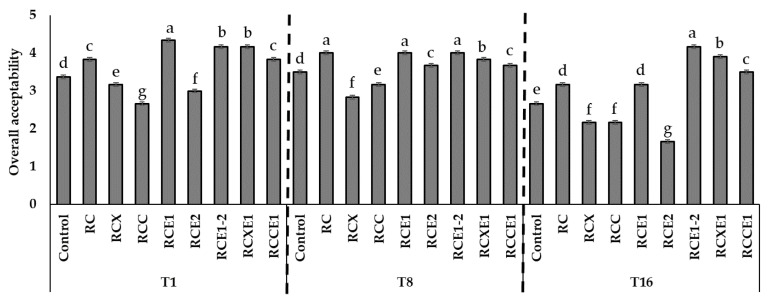
Average values of overall acceptability evaluated through panel test, carried out after 1 (T1), 8 (T8), and 16 (T16) days of storage at 4 °C on the Burrata cheeses made from whole milk and cream (Control); semi-skimmed milk and reduced-fat cream (RC); semi-skimmed milk and reduced-fat cream diluted with xanthan (RCX) or carrageenan (RCC); semi-skimmed milk added with exopolysaccharide producing starter E1 and reduced-fat cream (RCE1); semi-skimmed milk and reduced-fat cream added with exopolysaccharide producing starter E2 (RCE2); semi-skimmed milk and reduced-fat cream both added with E1 and E2 (RCE1-2); semi-skimmed milk added with E1 and reduced-fat cream diluted with xanthan (RCXE1) or carrageenan (RCCE1).Within the same time of analysis, bars labelled with the same letter represent not significantly (*p* > 0.05) different values.

All of the Burrata cheeses were subjected to a panel test, after 1, 8, and 16 days from manufacturing. After 1 day, RCE1, RCE1-2, and RCXE1 Burrata cheeses received average scores (on a 5-points scale) higher than 4 for overall acceptability (Figure 4). This attribute ranged between 3.2 and 3.8 for RC, RCX, RCCE1, and control cheeses. After 8 days, overall acceptability ranged from 2.8 (RCX) to 4.0 (RC, RCE1, RCE1-2). After 16 days, the highest (*p* < 0.05) score was attributed to RCE1-2 (4.2 on average). Scores of 3.9 and 3.5 were attributed to RCXE1 and RCCE1 Burrata cheeses for overall acceptability (Figure 4).

**Figure 5:**
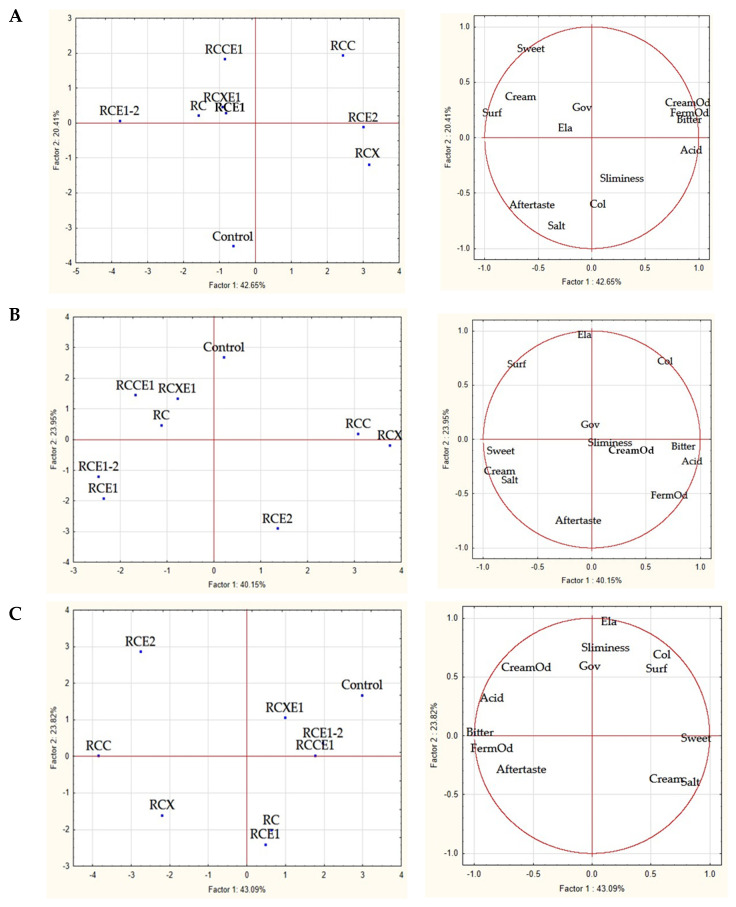
Scores and loading plots of the first and second principal components after Principal Component Analysis (PCA) based on the sensory attributes evaluated through panel test. (**A**) PCA carried out after 1 day; (**B**) PCA carried out after 8 days; (**C**) PCA carried out after 16 days of storage at 4 °C of the Burrata cheeses made from whole milk and cream (Control); semi-skimmed milk and reduced-fat cream (RC); semi-skimmed milk and reduced-fat cream diluted with xanthan (RCX) or carrageenan (RCC); semi-skimmed milk added with exopolysaccharide producing starter E1 and reduced-fat cream (RCE1); semi-skimmed milk and reduced-fat cream added with exopolysaccharide producing starter E2 (RCE2); semi-skimmed milk and reduced-fat cream both added with E1 and E2 (RCE1-2); semi-skimmed milk added with E1 and reduced-fat cream diluted with xanthan (RCXE1) or carrageenan (RCCE1).

The results of the other attributes (governing liquid transparency, color, surface appearance, elasticity, sliminess, cream milk odor, fermented milk odor, acid taste, bitter taste, sweet taste, cream milk taste, salty taste, and aftertaste) evaluated through the panel test are shown in Table S2 and were elaborated through Principal Component Analysis (PCA) (Figure 5). Regardless of time of analysis, two first components explained at least 63.06% of total variance. At 1 day, the negative segment of PC1 showed the loading of surface appearance (Surf), whereas the positive segment showed the loading of creamy (CreamOd) and fermented (FermOd) odors, and bitter taste. Sweet and salty taste showed the highest positive and negative, respectively, loads for PC2. The cheese variants were distributed in two groups: RC, RCE1, RCXE1, RCCE1, RCE1-2, and control Burrata cheeses fell in the left quadrants of the plane; the other variants fell in the right quadrants. Within the first group, the control showed many sensory differences, reporting the highest scores for salty taste and color. RCE1-2 was particularly appreciated for its good surface appearance (Figure 5A).

After 8 days of storage, the negative segment of PC1 showed the loading of sweet taste, whereas the positive segment showed the loading of bitter and acid tastes. Aftertaste and elasticity (Ela) showed the highest negative and positive, respectively, loads for PC2. Again, RC, RCE1, RCXE1, RCCE1, and RCE1-2 Burrata cheeses fell in the left quadrants of the plane, whereas the control and the other Burrata cheeses fell in the right quadrants. However, the group of cheeses in the right quadrants included two Burrata cheeses (RCC and RCX), judged as very similar, characterized by being bitter, acid and with a strong fermented milk odor. On the contrary, the two other Burrata cheese variants (control and RCE2) showed quite different sensory traits both each other and with respect to RCC and RCX (Figure 5B). 

After 16 days, the negative segment of PC1 showed the loading of bitter taste and fermented milk odor, whereas the positive segment showed the loading of sweet taste. Elasticity showed the highest positive load for PC2. RCCE1, RCXE1, RCE1-2, and control Burrata cheeses grouped in the fourth quadrant of the plane, being evaluated as sweet and elastic. RC and RCE1 fell in the third quadrant, whereas RCE2, RCX, and RCC fell in the first, second and at borderline between first and second quadrant, respectively. Bitter and acid taste and strong unpleasant aftertaste characterized RCE2, RCX, and RCC Burrata cheeses (Figure 5C).

### 3.5. Burrata Microbiome

**Figure 6:**
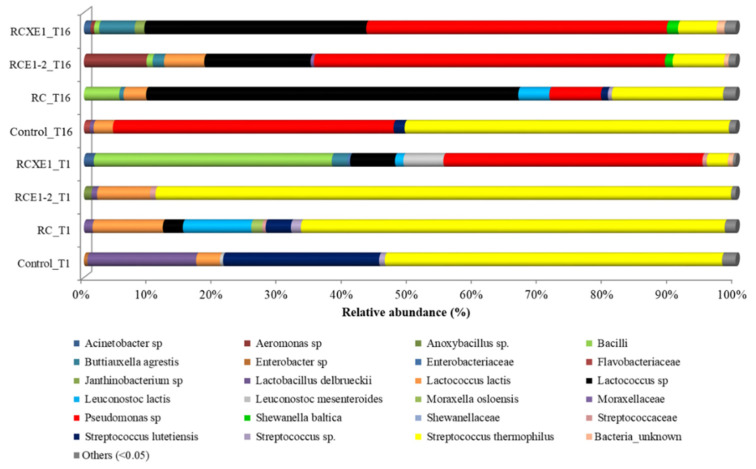
Relative abundance (%) of the main bacterial Operational Taxonomic Units (OTUs) assigned at the highest possible taxonomic level found after 1 (T1) and 16 (T16) days of storage at 4 °C, in the Burrata cheeses made from: whole milk and cream (Control); semi-skimmed milk and reduced-fat cream (RC); semi-skimmed milk and reduced-fat cream both added with E1 and E2 (RCE1-2); semi-skimmed milk added with E1 and reduced-fat cream diluted with xanthan (RCXE1). “Others” represent OTUs found at relative abundance less than 0.05%.

In order to understand the potential role of bacterial community in the sensory traits, the following four Burrata cheese variants were selected for analysis of bacterial microbiome after 1 and 16 days of storage: RCXE1 and RCE1-2, being the most preferred (based on the results from the panel test) Burrata cheeses; RC, being a reduced-fat cheese, exactly as RCXE1 and RCE1-2, but without additional EPS-producing starters and xanthan or carrageenan; control, being the traditional full-fat Burrata cheese. At 1 day, *Streptococcus thermophilus* was the most abundant bacterial species in the control, RC and RCE1-2 Burrata cheeses (Figure 6, Table S3). In the control, this Operational Taxonomic Unit (OTU) (relative abundance: 51.8%) was flanked by *Streptococcus lutetiensis* (23.9%), *Lactobacillus delbrueckii* (16.7%) and *Lactococcus lactis* (3.6%). RC Burrata cheese harbored, besides *S. thermophilus* (65%), *Lc. lactis* (10.8%), *Leuconostoc lactis* (10.5%), and, as minor OTUs, *Lactobacillus delbrueckii* (1.3%) and *Moraxella osloensis* (1.7%). In RCE1-2 Burrata cheese, besides *S. thermophilus* (88.3%), *Lc. lactis* (8.1%) was detected as sub-dominant. RCXE1 Burrata cheese harbored *Pseudomonas* sp. (39.7%) and *Bacilli* (36.5%) as dominant OTUs, and *Lactococcus* sp. (6.9%), *Leuconostoc mesenteroides* (6.1%), and *S. thermophilus* (3%) as sub-dominant/minor OTUs (Figure 6, Table S3).

After 16 days of storage, *S. thermophilus* was still the dominant OTU (relative abundance: 49.7%) in the control cheese, but it was flanked by *Pseudomonas* sp. (43.1%). The control Burrata cheese also harbored *Lc. lactis* (3%) and *S. lutetiensis* (1.7%) as sub-dominant OTUs. RC harbored *Lactococcus* sp. (57.1%), *S. thermophilus* (17.1%), *Pseudomonas* sp. (7.9%), *Leuc. lactis* (4.8%), Bacilli (5.4%), and *Lc. lactis* (3.5%). RCXE1 and RCE1-2 showed quite a similar profile of bacterial biota, with dominance of *Pseudomonas* sp. (46.1–53.9%), followed by *Lactococcus* sp. (14–34%) and *S. thermophilus* (5.9–7.9%). These two Burrata cheeses shared also some less abundant OTUs, such as *Shewanella baltica* and Bacilli. The only differences found in these cheeses were for *Aeromonas* sp. and *Buttiauxella agrestis*, found in RCE1-2 at higher and lower (respectively) relative abundance than in RCXE1 (Figure 6, Table S3).

### 3.6. Correlations between Microbiota and Biochemical Characteristics of Burrata Cheeses

**Figure 7:**
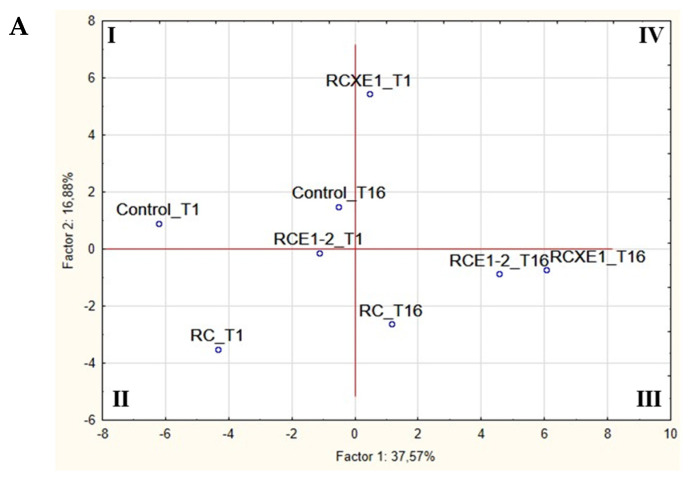

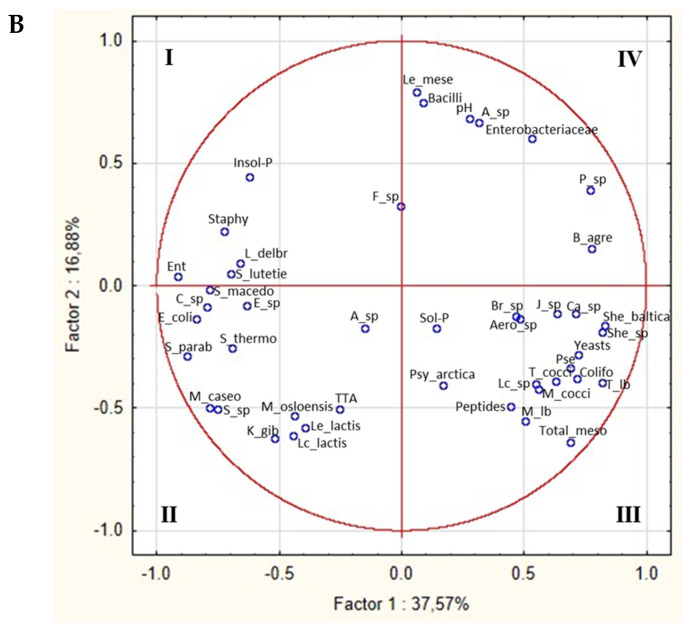
Scores (**A**) and loading (**B**) plots of first and second principal components after Principal Component Analysis based on microbiological (cell densities, OTU relative abundance) and biochemical (pH, TTA, concentrations of proteins in pH 4.6-soluble and -insoluble fraction, concentration of peptides in pH 4.6-soluble fraction) characteristics determined after 1 (T1) and 16 (T16) days of storage at 4 °C on the Burrata cheeses made from: whole milk and cream (Control); semi-skimmed milk and reduced-fat cream (RC); semi-skimmed milk and reduced-fat cream both added with E1 and E2 (RCE1-2); semi-skimmed milk added with E1, and reduced-fat cream diluted with xanthan (RCXE1). Total titratable acidity, TTA; total mesophilic microorganisms, Total_meso; mesophilic lactobacilli, M_lb; thermophilic lactobacilli, T_lb; mesophilic cocci, M_cocci; thermophilic cocci, T_cocci; enterococci, Ent; presumptive *Pseudomonas* sp., Pse; staphylococci, Staphy; coliforms, Colifo; proteins in insoluble fraction, Inso-P; proteins in soluble fraction, Sol-P; *Chryseobacterium* sp., C_sp; *Flavobacterium* sp., F_sp; *Anoxybacillus* sp., A_sp; *Brochothrix* sp., Br_sp; *Kurthia gibsonii*, K_gib; *Macrococcus caseolyticus*, M_caseo; *Carnobacterium* sp., Ca_sp; *Lactobacillus delbrueckii*, L_delbr; *Leuconostoc lactis*, Le_lactis; *Leuconostoc mesenteroides*, Le_mese; *Lactococcus lactis*, Lc_lactis; *Lactococcus* sp., Lc_sp; *Streptococcus lutetiensis*, S_lutetie; *Streptococcus macedonicus*, S_macedo; *Streptococcus parauberis*, S_parab; *Streptococcus thermophilus*, (S_thermo); *Streptococcus* sp. (S_sp); *Janthinobacterium* sp., J_sp; *Aeromonas* sp., Aero_sp; *Psychromonas arctica*, Psy_arctica; *Shewanella baltica*, She_baltica; *Buttiauxella agrestis*, B_agre; *Enterobacter* sp., E_sp; *Escherichia coli*, E_coli; *Acinetobacter* sp., A_sp; *Moraxella osloensis*, M_osloensis; *Pseudomonas* sp., P_sp.

Results from microbiological (cell densities of all of the microbial groups, relative abundance of bacterial OTUs) and biochemical characterization (pH, TTA, concentrations of proteins and peptides in the cheese extracts) of the two most preferred reduced-fat Burrata cheeses (RCXE1 and RCE1-2), of the reduced-fat cheese RC, and of the traditional full-fat Burrata cheese (control) were elaborated through PCA, in order to better estimate the influence of variables on the quality of cheeses (Figure 7). At day 1, RCE1-2 and RC Burrata cheeses were clearly differentiated from RCXE1 and control Burrata cheeses (Figure 7A). The two latter cheeses were also separated one from each other. After 16 days of storage, all of the reduced-fat Burrata cheeses fell in the third quadrant of the plane, thus being clearly differentiated from all of the other cheeses, including the control analyzed at 16 days. Among the variables, thermophilic lactobacilli and enterococci showed high positive and negative loadings on PC1, respectively (Figure 7B).

Several negative (*r* < −0.7) and positive (*r* > 0.7) correlations between variables were found (Figure S2). For instance, pH was negatively correlated with *Leuc. lactis* and *Streptococcus* sp. *S. thermophilus* was negatively correlated with the OTUs *Enterobacteriaceae* and *Pseudomonas* sp. and positively with *Lc. lactis*. Positive correlations were found between *Macrococcus caseolyticus* and *Streptococcus* sp., *Streptococcus parauberis*, *Leuc. lactis,* and *Lc. lactis*. Cell densities of thermophilic lactobacilli were negatively correlated with enterococci and staphylococci.

### 3.7. Consumer Test

**Figure 8:**
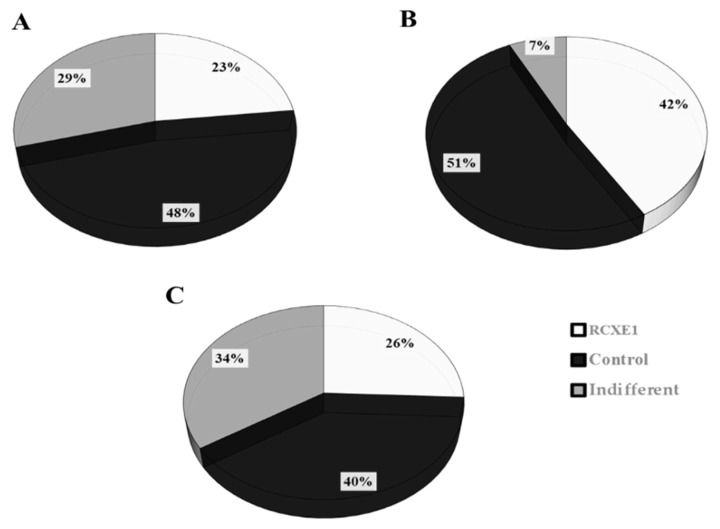
Percentages of consumers expressing compared judgment about appearance (**A**), texture (**B**), and odor (**C**) between the traditional full-fat Burrata cheese (Control) and the reduced-fat Burrata cheese produced from semi-skimmed milk added with E1 and reduced-fat cream diluted with xanthan (RCXE1). Compared judgment was expressed using one of the following phrases: (i) preference for Control; (ii) preference for RCXE1; (iii) no difference between the two cheeses (indifferent).

RCXE1 was subjected, along with control Burrata cheese, to a consumer test, because it contained fat concentration lower (ca. 50%) than the control and had been (together with RCE1-2) the most preferred cheese, on the basis of the results from the panel test. Among the consumers participating in the test, most (92.7%) were occasional consumers of Burrata cheese, whereas the remaining 7.3% declared to eat Burrata at least once a week. The data processing highlighted that 53.7% of the consumers did not distinguish, at first sight and taste, the reduced-fat Burrata cheese (RCXE1) from the control. After disclosure of the Burrata cheese variants, although 48% of consumers preferred the control in terms of appearance, 23% preferred the reduced-fat Burrata cheese, and 29% did not perceive any difference between the two cheese variants (Figure 8A). In terms of texture, 51% of consumers preferred the control, 42% preferred the reduced-fat cheese, and 7% perceived the two cheese as not different (Figure 8B). In regards to odor, 40% of participants preferred the traditional Burrata cheese, 26% preferred the reduced-fat Burrata cheese, and 34% did not perceive any difference (Figure 8C). Lastly, the average acceptability scores were 8.0 ± 0.80 for traditional Burrata cheese and 7.0 ± 1.40 for RCXE1 reduced-fat Burrata.

## 4. Discussion

Reduction of dietary fat intake is one of the main recommendations by the World Health Organization (WHO) [56,57,58]. Cheeses are among the main vehicles of dietary fat but, at the same time, represent a frequent and pleasant dietary choice for many consumers [59,60]. Therefore, more and more cheesemakers and researchers are interested in strategies to produce low-fat or reduced-fat cheese. However, production of such cheeses is a challenge, because fat has a pivotal role in structure and flavor of cheese [30,34,61,62]. Consumers perceive these products as different from their full-fat counterpart because excessively dry, firm or difficult to chew and with atypical flavor [26,29]. Burrata cheese popularity is growing, but it can contain up to 60% of fat [1]. Thus, the task of producing a reduced-fat Burrata is more challenging than for other cheeses. To our knowledge, in the only study aiming to develop a strategy for reducing fat in Burrata, partially skimmed milk and carob seeds suspension as fat replacer were combined, obtaining a reduced-fat Burrata cheese judged as having good sensory characteristics, but perceived different from the full-fat Burrata cheese [1]. This study used semi-skimmed milk, reduced-fat cream and two commercially available fat replacers, namely carrageenan and xanthan gums, alone or in combination with EPS-synthesizing lactic acid bacteria (LAB), to improve the sensory quality of reduced-fat Burrata cheese. Besides seven variants of reduced-fat cheese, a further reduced-fat Burrata, obtained by combining just semi-skimmed milk and reduced-fat cream, and a full-fat traditional Burrata (used as the control) were included in the experimental design. Burrata cheeses were manufactured at the same dairy plant, under the same biotic (house microbiota) and abiotic (e.g., acidification, stretching temperature, and salt concentration) selection pressures, which may affect cheese microbiota [63,64].

All of the reduced-fat Burrata cheeses were characterized by lower lipid concentration and higher moisture than the control, in agreement with previous studies [1,39,65]. The inverse correlation between lipid and moisture could be partly explained by the presence of xanthan, carrageenan or EPS putatively synthesized by the bacterial cultures (streptococci or lactococci or both) added in milk or cream. Indeed, these compounds act as fat replacers and, as such, they are able to bind water, thus resulting in higher moisture in cheese [65]. Post-acidification during storage occurred for the Burrata cheeses containing carrageenan (RCC) or xanthan (RCX) as fat replacers or produced by inoculating cream with a commercial EPS-synthesizing culture consisting of two strains of *Lactococcus lactis* (RCE2). This could be related to higher (order of magnitude: 8 log cfu/g) cell density of mesophilic cocci found in those cheeses after 16 days of storage. Mesophilic cocci (e.g., lactococci and *Leuconostoc*) also include psychrotrophic bacteria, which are able to keep on acidifying milk and dairy products during refrigerated storage [66,67]. It may be hypothesized that psychrotrophic mesophilic cocci, contributing to post-acidification of the Burrata cheeses analyzed in this study, belong to dairy house microbiota. Indeed, in a previous study on Burrata cheese, manufactured at the same dairy plant, *Lc. lactis* and *Leuconostoc lactis* were detected as components of the core microbiota of cheese, probably originating from cheese-making environment [4].

In this study, culture-dependent microbiological characterization of the Burrata cheeses showed that mesophilic and thermophilic cocci, although showing cheese variant-depending dynamics during storage, were dominant microbial groups. Presumptive *Pseudomonas* sp. flanked these two groups during storage, reaching cell densities higher than 6 log CFU g^−1^. These results were in agreement with a previous study performed on Burrata in the same dairy plant [4]. Overall, mesophilic and thermophilic lactobacilli increased during storage and represented other dominant microbial groups, although with a cell density, on average, 1 log cycle lower than coccus-shaped LAB. This result was in contrast with the above-mentioned previous study, wherein rod-shaped LAB decreased during storage and belonged to sub-dominant microbiota [4]. In our experimental Burrata cheeses, sub-dominant cultivable microbiota was composed of enterococci, coliforms and yeasts.

Level of proteolysis showed little variations, depending on the Burrata cheese variant. However, overall proteins and peptides in the pH 4.6-soluble fraction increased during storage. As assessed through urea-PAGE and spectrophotometric determination, proteins in the pH 4.6-insoluble fraction were subjected to limited hydrolysis during storage, in agreement with previous studies [1,4].

The panel test showed that the addition of the EPS-producing *Streptococcus thermophilus* in milk, in combination with either EPS-producing lactococci in cream (RCE1-2) or xanthan gum suspension (RCXE1), improved the overall acceptability of the Burrata cheeses. Even after 16 days of storage, RCE1-2 and RCXE1 were appreciated especially for their taste and texture. On the contrary, RCE2, RCX, RCC Burrata cheeses were judged as bitter and acid and with unpleasant aftertaste, being consistent with the post-acidification that characterized these cheeses. Therefore, bacterial microbiomes of RCE1-2 and RCXE1, as well as of the control and reduced-fat Burrata cheese not added with EPS-producing LAB and/or gums (RC), were described through 16S-targeted metagenomic analysis. A core microbiota, including *S. thermophilus*, *Streptococcus lutetiensis*, *Lc. lactis*, *Lactococcus* sp., *Leuconostoc lactis*, *Lactobacillus delbrueckii*, and *Pseudomonas* sp., characterized all of the Burrata cheeses, although with differences of relative abundance among the cheeses. *S. thermophilus*, used as starter E1 in the RCE1, RCE1-2, RCXE1, and RCCE1 Burrata cheeses, was one of the dominant OTUs in the control, RC, and RCE1-2 Burrata cheeses. Dominance of *S. thermophilus* in Burrata cheeses manufactured without starter E1 is probably due to contamination from dairy environment, in agreement with previous studies on cheeses manufactured at the same dairy plant used in this study [4,68,69]. Notwithstanding the high relative abundance of *Pseudomonas* sp. in three out of four Burrata cheeses, no defect, such as bitter taste and discoloration, was found at the end of storage. This could be explained by the species- and strain-specific spoilage ability by *Pseudomonas* sp. [70].

Multivariate statistical analysis showed that cell density of thermophilic lactobacilli could explain the distribution of Burrata cheeses. This bacterial group, with pro-technological role in fresh pasta filata cheese [71], was found in the RCE1-2 and RCXE1 at higher number (6.8 log CFU g^−1^) than control (4.3 log CFU g^−1^) and RC (5.8 log CFU g^−1^) Burrata cheeses. The positive correlation between pH and two coccus-shaped LAB OTU (*Leuc. lactis* and *Streptococcus* sp.) was in agreement with the results of culture-dependent analysis. Some positive correlations (e.g., *Leuc. lactis*, *Lc. lactis*, and *Macrococcus caseolyticus*) were in agreement with co-occurrence patterns observed for Caciotta cheese manufactured at the same dairy plant [69].

The consumer test indicated that more than 50% of consumers did not distinguish the traditional full-fat (control) from RCXE1 Burrata cheese. Fat concentration in the latter cheese was about 50% lower than the control, allowing to label this product as “reduced-fat” [72]. After the two variants of Burrata cheese were disclosed to consumers, a majority of them preferred the control in terms of appearance, texture and odor. This is not so surprising, given that sensory tests are affected by several biases [73]. Although we used a non-forced choice preference testing, we may hypothesize that the variable “sample disclosure” probably affected consumers’ preference, depending on the individual aptitude towards traditional or novel foods.

This study showed that the combination of semi-skimmed milk inoculated with EPS-synthesizing streptococci and reduced-fat cream diluted with xanthan gum suspension led to obtain a reduced-fat Burrata cheese with valuable sensory traits and very similar to the traditional full-fat counterpart.

## Figures and Tables

**Table 1:** Gross composition (g/100 g) *, determined after 1 day of manufacture, of the experimental Burrata cheeses ^†^.

Burrata Cheese Variant	Carbohydrates	Proteins	Total Fat	Moisture	Sodium
**Control**	1.2 ± 0.06 ^a^	10.4 ± 0.03 ^c^	21.6 ± 0.05 ^a^	65.4 ± 0.90 ^c^	0.50 ± 0.02 ^a^
**RC**	0.8 ± 0.03 ^b^	11.6 ± 0.01 ^a^	16.0 ± 0.02 ^b^	70.5 ± 1.30 ^b^	0.14 ± 0.02 ^c^
**RCX**	1.1 ± 0.09 ^a^	10.5 ± 0.03 ^bc^	10.0 ± 0.05 ^e^	72.0 ± 1.40 ^ab^	0.16 ± 0.02 ^bc^
**RCC**	1.2 ± 0.06 ^a^	10.0 ± 0.01 ^d^	10.6 ± 0.03 ^e^	71.5 ± 1.20 ^b^	0.20 ± 0.02 ^b^
**RCE1**	0.8 ± 0.05 ^b^	11.0 ± 0.03 ^b^	13.0 ± 0.05 ^d^	70.3 ± 1.40 ^b^	0.14 ± 0.03 ^c^
**RCE2**	1.0 ± 0.05 ^a^	11.2 ± 0.02 ^b^	14.5 ± 0.04 ^c^	70.4 ± 1.20 ^b^	0.20 ± 0.01 ^b^
**RCE1-2**	1.1 ± 0.06 ^a^	11.0 ± 0.01 ^b^	14.5 ± 0.05 ^c^	72.3 ± 1.40 ^ab^	0.20 ± 0.02 ^b^
**RCXE1**	1.2 ± 0.05 ^a^	10.6 ± 0.01 ^c^	11.0 ± 0.04 ^e^	75.7 ± 1.20 ^a^	0.16 ± 0.03 ^bc^
**RCCE1**	1.2 ± 0.07 ^a^	10.6 ± 0.03 ^c^	11.5 ± 0.05 ^e^	73.6 ± 1.20 ^ab^	0.16 ± 0.03 ^bc^

* Within the same column, values sharing one or more superscript letters (a–e) are not significantly (*p* > 0.05) different. ^†^ Control, cheese made from whole milk and cream; RC, cheese made from semi-skimmed milk and skimmed cream; RCX, cheese made from semi-skimmed milk and reduced-fat cream diluted with xanthan; RCC, cheese made from semi-skimmed milk and reduced-fat cream diluted with carrageenan; RCE1, cheese made from semi-skimmed milk added with exopolysaccharide producing starter E1 and reduced-fat cream; RCE2, cheese made from semi-skimmed milk, reduced-fat cream added with exopolysaccharide producing starter E2; RCE1-2, cheese made from semi-skimmed milk and reduced-fat cream both added with E1 and E2; RCXE1, cheese made from semi-skimmed milk added with E1 and reduced-fat cream diluted with xanthan; RCCE1, cheese made from semi-skimmed milk added with E1 and reduced-fat cream diluted with carrageenan.

## Supplementary Materials

Supplementary materials can be found at https://www.mdpi.com/2076-2607/8/10/1618/s1. Figure S1: Concentration of total peptides, determined after 1 (T1) and 16 (T16) days of storage at 4 °C, in the pH 4.6-soluble fraction of Burrata cheeses made from whole milk and cream (Control); semi-skimmed milk and reduced-fat cream (RC); semi-skimmed milk and reduced-fat cream diluted with xanthan (RCX) or carrageenan (RCC); semi-skimmed milk added with exopolysaccharide producing starter E1 and reduced-fat cream (RCE1); semi-skimmed milk and reduced-fat cream added with exopolysaccharide producing starter E2 (RCE2); semi-skimmed milk and reduced-fat cream both added with E1 and E2 (RCE1-2); semi-skimmed milk added with E1 and reduced-fat cream diluted with xanthan (RCXE1) or carrageenan (RCCE1). Within the same sample, bars labelled with the same letter represent not significantly (*p* > 0.05) different values. Figure S2: Correlations based on microbiological (cell densities, OTU relative abundance) and biochemical (pH, TTA, concentrations of proteins in pH 4.6-soluble and –insoluble fraction, concentration of peptides in pH 4.6-soluble fraction) characteristics determined after 1 and 16 days of storage at 4 °C on the burrata cheeses made from: whole milk and cream (Control); semi-skimmed milk and reduced-fat cream (RC); semi-skimmed milk and reduced-fat cream both added with E1 and E2 (RCE1-2); semi-skimmed milk added with E1 and reduced-fat cream diluted with xanthan (RCXE1). The colors of the scale bar denote the nature of the correlation, with 1.00 indicating a perfectly positive correlation (red) and −1.00 indicating a perfectly negative correlation (green). Asterisk indicates significant correlations (FDR < 0.05). Table S1 Cheese-making ingredients used for the manufacturing of control and experimental (RC, RCX, RCC, RCE1, RCE2, RCE1-2, RCXE1, RCCE1) Burrata cheeses. Table S2 Average scores (from 1, lowest, to 5, highest) of sensory attributes resulting from the panel tests carried out on control and experimental (RC, RCX, RCC, RCE1, RCE2, RCE1-2, RCXE1, RCCE1) Burrata cheeses *^a^* after 1 (T1), 8 (T8) and 16 (T16) days of storage at 4 °C. Table S3 Relative abundance (%) *^a^* of bacterial species found through 16S metagenetic analysis of DNA extracted after 1 (T1) and 16 (T16) days of storage at 4 °C, in the experimental Burrata cheeses *^b^*.
