# Supplementary material for: Use of Exopolysaccharide-Synthesizing Lactic Acid Bacteria and Fat Replacers for Manufacturing Reduced-Fat Burrata Cheese: Microbiological Aspects and Sensory Evaluation
Source: Microorganisms. 2020 Oct 21;8(10):1618. doi: 10.3390/microorganisms8101618 (PMC7588969; doi:10.3390/microorganisms8101618)
Supplement: Supplementary file 1 [file microorganisms-08-01618-s001.zip › Costantino et al._supplementary tables_rev1.docx]

**TABLE S1** Cheese-making ingredients used for the manufacturing of control and experimental (RC, RCX, RCC, RCE1, RCE2, RCE1-2, RCXE1, RCCE1) Burrata cheeses.

|  | Whole Milk | Semi-skimmed milk | Semi-skimmed milk inoculated with the starter E1*^a^* | Cream | Reduced-fat cream | Reduced-fat cream inoculated with starter E2*^b^* | Reduced-fat cream  diluted with xanthan | Reduced-fat cream  diluted with carrageenan |
| --- | --- | --- | --- | --- | --- | --- | --- | --- |
| **Control** | x |  |  | x |  |  |  |  |
| **RC** |  | x |  |  | x |  |  |  |
| **RCX** |  | x |  |  |  |  | x |  |
| **RCC** |  | x |  |  |  |  |  | x |
| **RCE1** |  |  | x |  | x |  |  |  |
| **RCE2** |  | x |  |  |  | x |  |  |
| **RCE1-2** |  |  | x |  |  | x |  |  |
| **RCXE1** |  |  | x |  |  |  | x |  |
| **RCCE1** |  |  | x |  |  |  |  | x |

*^a^* Semi-skimmed milk directly inoculated (6% wt/vol) with the exopolysaccharide producing starter E1 (*Streptococcus thermophilus*).
*^b^* Reduced-fat cream directly inoculated (3% wt/vol) with the exopolysaccharide producing starter E2 (*Lactococcus lactis* subsp. *lactis* and *Lactococcus lactis* subsp. *cremoris*).

**TABLE S2** Average scores (from 1, lowest, to 5, highest) of sensory attributes resulting from the panel tests carried out on control and experimental (RC, RCX, RCC, RCE1, RCE2, RCE1-2, RCXE1, RCCE1) Burrata cheeses*^a^* after 1 (T1), 8 (T8) and 16 (T16) days of storage at 4 °C.

|  | Burrata cheese variant | Governing liquid transparency | Color | Surface appearance | Sliminess | Elasticity | Fermented  milk odor | Cream  milk odor | Salty taste | Acid taste | Bitter taste | Sweet taste | Cream milk taste | Aftertaste |
| --- | --- | --- | --- | --- | --- | --- | --- | --- | --- | --- | --- | --- | --- | --- |
| **T1** | **Control** | 2.1 | 4.4 | 3.8 | 1.8 | 3.6 | 1.4 | 2.6 | 3.9 | 2.0 | 1.0 | 2.8 | 3.4 | 3.8 |
|  | **RC** | 2.2 | 3.3 | 4.5 | 1.3 | 2.2 | 1.3 | 2.3 | 3.2 | 1.3 | 1.0 | 3.2 | 3.3 | 3.5 |
|  | **RCX** | 2.2 | 3.7 | 3.2 | 1.8 | 2.0 | 1.8 | 3.7 | 3.2 | 3.0 | 1.2 | 2.8 | 3.0 | 3.5 |
|  | **RCC** | 2.0 | 3.5 | 3.2 | 1.3 | 2.8 | 2.0 | 3.7 | 2.8 | 2.3 | 1.2 | 3.3 | 3.5 | 2.8 |
|  | **RCE1** | 1.7 | 3.7 | 3.8 | 1.8 | 2.8 | 1.7 | 3.0 | 3.2 | 1.3 | 1.0 | 3.3 | 4.0 | 3.3 |
|  | **RCE2** | 3.7 | 3.8 | 3.2 | 1.8 | 2.3 | 2.3 | 3.5 | 3.0 | 2.7 | 1.2 | 3.0 | 3.3 | 3.3 |
|  | **RCE1-2** | 3.2 | 3.7 | 4.8 | 1.3 | 2.3 | 1.0 | 2.0 | 3.5 | 1.2 | 1.0 | 3.7 | 4.0 | 3.8 |
|  | **RCXE1** | 3.3 | 3.3 | 3.8 | 2.2 | 3.5 | 1.3 | 3.2 | 2.8 | 1.7 | 1.0 | 3.2 | 4.0 | 3.7 |
|  | **RCCE1** | 3.2 | 3.8 | 4.5 | 1.2 | 3.8 | 1.5 | 3.2 | 2.7 | 1.8 | 1.0 | 3.5 | 3.5 | 3.2 |
| **T8** | **Control** | 2.2 | 4.0 | 4.3 | 2.5 | 3.8 | 1.3 | 3.5 | 2.7 | 1.7 | 1.2 | 3.7 | 3.5 | 2.7 |
|  | **RC** | 2.2 | 3.3 | 3.8 | 2.0 | 3.7 | 1.5 | 2.3 | 3.0 | 1.3 | 1.2 | 3.5 | 3.5 | 3.7 |
|  | **RCX** | 2.8 | 3.8 | 3.2 | 2.3 | 3.5 | 2.3 | 3.5 | 2.5 | 2.8 | 1.5 | 3.0 | 3.3 | 3.5 |
|  | **RCC** | 2.7 | 3.7 | 3.2 | 2.2 | 3.3 | 2.2 | 3.0 | 2.5 | 2.8 | 1.3 | 3.2 | 3.0 | 3.3 |
|  | **RCE1** | 1.7 | 2.7 | 3.8 | 2.3 | 2.7 | 1.7 | 2.8 | 3.0 | 1.5 | 1.2 | 4.3 | 4.2 | 3.7 |
|  | **RCE2** | 3.3 | 3.3 | 3.0 | 2.7 | 2.2 | 2.0 | 3.5 | 2.8 | 2.8 | 1.2 | 3.5 | 3.7 | 4.2 |
|  | **RCE1-2** | 3.3 | 3.0 | 3.8 | 2.2 | 3.2 | 1.8 | 3.3 | 3.2 | 1.0 | 1.0 | 4.0 | 4.2 | 3.7 |
|  | **RCXE1** | 3.8 | 3.7 | 3.8 | 2.5 | 3.5 | 1.3 | 2.7 | 2.5 | 1.7 | 1.0 | 4.0 | 3.7 | 3.3 |
|  | **RCCE1** | 3.8 | 3.3 | 4.7 | 2.3 | 4.2 | 1.3 | 3.2 | 2.8 | 1.5 | 1.0 | 3.5 | 3.8 | 3.7 |
| **T16** | **Control** | 1.8 | 4.0 | 3.0 | 3.0 | 3.5 | 1.2 | 2.3 | 2.5 | 1.8 | 1.0 | 4.0 | 2.8 | 2.3 |
|  | **RC** | 2.0 | 3.2 | 3.0 | 2.7 | 2.5 | 2.0 | 2.0 | 2.7 | 2.0 | 1.2 | 3.5 | 3.3 | 4.0 |
|  | **RCX** | 2.8 | 3.3 | 2.3 | 2.2 | 2.3 | 2.2 | 2.3 | 2.3 | 2.8 | 2.2 | 3.0 | 3.0 | 3.6 |
|  | **RCC** | 2.2 | 2.7 | 2.3 | 2.3 | 3.3 | 2.2 | 3.0 | 1.8 | 2.8 | 2.2 | 3.0 | 2.7 | 3.7 |
|  | **RCE1** | 1.5 | 3.0 | 2.7 | 2.3 | 2.3 | 1.7 | 2.5 | 2.7 | 1.7 | 1.3 | 3.3 | 3.5 | 3.5 |
|  | **RCE2** | 3.5 | 3.7 | 3.3 | 3.2 | 3.5 | 2.2 | 2.8 | 2.0 | 3.7 | 1.8 | 2.7 | 2.7 | 3.8 |
|  | **RCE1-2** | 3.8 | 3.7 | 3.2 | 2.7 | 3.3 | 1.8 | 2.5 | 2.7 | 1.3 | 1.0 | 3.7 | 3.7 | 3.5 |
|  | **RCXE1** | 4.0 | 3.5 | 3.3 | 2.5 | 3.5 | 1.7 | 2.7 | 2.3 | 1.3 | 1.3 | 3.3 | 3.5 | 3.3 |
|  | **RCCE1** | 2.8 | 3.5 | 3.7 | 2.2 | 3.3 | 1.7 | 2.5 | 2.7 | 1.3 | 1.0 | 3.3 | 3.3 | 3.3 |

*^a^* Control, cheese made from whole milk and cream; RC, cheese made from semi-skimmed milk and skimmed cream; RCX, cheese made from semi-skimmed milk and reduced-fat cream diluted with xanthan; RCC, cheese made from semi-skimmed milk and reduced-fat cream diluted with carrageenan; RCE1, cheese made from semi-skimmed milk added with exopolysaccharide producing starter E1 and reduced-fat cream; RCE2, cheese made from semi-skimmed milk, reduced-fat cream added with exopolysaccharide producing starter E2; RCE1-2, cheese made from semi-skimmed milk and reduced-fat cream both added with E1 and E2; RCXE1, cheese made from semi-skimmed milk added with E1 and reduced-fat cream diluted with xanthan; RCCE1, cheese made from semi-skimmed milk added with E1 and reduced-fat cream diluted with carrageenan.

**TABLE S3** Relative abundance (%)*^a^* of bacterial species found through 16S metagenetic analysis of DNA extracted after 1 (T1) and 16 (T16) days of storage at 4 °C, in the experimental Burrata cheeses*^b^*.

|  |  | **T1** | | | | **T16** | | | |
| --- | --- | --- | --- | --- | --- | --- | --- | --- | --- |
| **Phylum/Family** | **Species** | **Control** | **RC** | **RCE1-2** | **RCXE1** | **Control** | **RC** | **RCE1-2** | **RCXE1** |
| Bacteroidetes/ Flavobacteriaceae | *Chryseobacterium* sp. | 0.16^a^ | 0.08^b^ | 0.00^c^ | 0.00^c^ | 0.02^c^ | 0.00^c^ | 0.00^c^ | 0.00^c^ |
|  | *Flavobacterium* sp. | 0.00^c^ | 0.00^c^ | 0.00^c^ | 0.12^b^ | 0.86^a^ | 0.01^c^ | 0.03^c^ | 0.03^c^ |
| Firmicutes/Bacillaceae | *Anoxybacillus* sp. | 0.12^c^ | 0.23^b^ | 1.14^a^ | 0.02^d^ | 0.01^d^ | 0.14^c^ | 0.12^c^ | 0.12^c^ |
| Firmicutes/Listeriaceae | *Brochothrix* sp. | 0.00^b^ | 0.01^b^ | 0.00^b^ | 0.06^b^ | 0.00^b^ | 0.10^b^ | 1.95^a^ | 0.02^b^ |
| Firmicutes/Planococcaceae | *Kurthia gibsonii* | 0.06^bc^ | 0.35^a^ | 0.02^c^ | 0.00^c^ | 0.00^c^ | 0.11^b^ | 0.00^c^ | 0.00^c^ |
| Firmicutes/Staphylococcaceae | *Macrococcus caseolyticus* | 0.08^b^ | 0.13^c^ | 0.03^c^ | 0.00^d^ | 0.02^cd^ | 0.03^c^ | 0.00^d^ | 0.00^d^ |
| Firmicutes/Carnobacteriaceae | *Carnobacterium* sp. | 0.00^b^ | 0.00^b^ | 0.00^b^ | 0.01^b^ | 0.01^b^ | 0.01^b^ | 0.02^b^ | 0.12^a^ |
| Firmicutes/Lactobacillaceae | *Lactobacillus delbrueckii* | 16.67^a^ | 1.33^b^ | 0.87^bc^ | 0.01^c^ | 0.61^bc^ | 0.19^c^ | 0.26^c^ | 0.08^c^ |
| Firmicutes/Leuconostocaceae | *Leuconostoc lactis* | 0.48^c^ | 10.49^a^ | 0.01^c^ | 1.29^c^ | 0.33^c^ | 4.77^b^ | 0.03^c^ | 0.11^c^ |
|  | *Leuconostoc mesenteroides* | 0.02^b^ | 0.03^b^ | 0.01^b^ | 6.15^a^ | 0.02^b^ | 0.14^b^ | 0.12^b^ | 0.11^b^ |
| Firmicutes/Streptococcaceae | *Lactococcus lactis* | 3.63^d^ | 10.77^a^ | 8.11^b^ | 0.33^e^ | 2.95^d^ | 3.47^d^ | 6.19^c^ | 0.12^e^ |
|  | *Lactococcus* sp. | 0.12^e^ | 3.08^de^ | 0.05^e^ | 6.89^d^ | 0.09^e^ | 57.12^a^ | 14.15^c^ | 34.06^b^ |
|  | *Streptococcus lutetiensis* | 23.95^a^ | 3.88^b^ | 0.26^e^ | 0.02^e^ | 1.69^c^ | 1.06^d^ | 0.05^e^ | 0.03^e^ |
|  | *Streptococcus macedonicus* | 0.24^a^ | 0.07^b^ | 0.05^b^ | 0.00^b^ | 0.03^b^ | 0.01^b^ | 0.00^b^ | 0.00^b^ |
|  | *Streptococcus parauberis* | 0.10^a^ | 0.10^a^ | 0.03^bc^ | 0.01^bc^ | 0.04^b^ | 0.03^bc^ | 0.00^c^ | 0.02^bc^ |
|  | *Streptococcus thermophilus* | 51.76^c^ | 65.04^b^ | 88.31^c^ | 3.23^f^ | 49.76^c^ | 17.08^d^ | 7.88^e^ | 5.91^ef^ |
|  | *Streptococcus* sp. | 0.87^b^ | 1.54^a^ | 0.46^cd^ | 0.24^de^ | 0.10^e^ | 0.60^bc^ | 0.10^e^ | 0.07^e^ |
| Firmicutes/Others | Bacilli_others | 0.02^de^ | 0.03^de^ | 0.03^de^ | 36.53^a^ | 0.02^d^ | 5.42^b^ | 0.93^c^ | 0.78^cd^ |
| Proteobacteria/Oxalobacteraceae | *Janthinobacterium* sp. | 0.00^c^ | 0.00^c^ | 0.00^c^ | 0.03^bc^ | 0.00^c^ | 0.07^b^ | 0.07^b^ | 1.50^a^ |
| Proteobacteria/Aeromonadaceae | *Aeromonas* sp. | 0.09^b^ | 0.07^b^ | 0.01^b^ | 0.03^b^ | 0.01^b^ | 0.03^b^ | 9.60^a^ | 0.57^b^ |
| Proteobacteria/Psychromonadaceae | *Psychromonas arctica* | 0.00^b^ | 0.00^b^ | 0.00^b^ | 0.00^b^ | 0.00^b^ | 0.11^a^ | 0.01^b^ | 0.00^b^ |
| Proteobacteria/Shewanellaceae | *Shewanella baltica* | 0.00^c^ | 0.00^c^ | 0.00^c^ | 0.11^c^ | 0.00^c^ | 0.15^bc^ | 1.19^b^ | 1.73^a^ |
|  | *Shewanella* sp. | 0.00^c^ | 0.00^c^ | 0.00^c^ | 0.00^c^ | 0.00^c^ | 0.02^c^ | 0.29^b^ | 0.39^a^ |
| Proteobacteria/Enterobacteriaceae | *Buttiauxella agrestis* | 0.01^e^ | 0.01^e^ | 0.00^e^ | 2.37^b^ | 0.01^e^ | 0.65^d^ | 1.75^c^ | 5.45^a^ |
|  | *Enterobacter* sp. | 0.55^a^ | 0.28^b^ | 0.06^d^ | 0.08^cd^ | 0.05^d^ | 0.04^d^ | 0.05^d^ | 0.19^bc^ |
|  | *Escherichia coli* | 0.08^a^ | 0.06^ab^ | 0.01^bc^ | 0.00^c^ | 0.03^abc^ | 0.00^c^ | 0.01^c^ | 0.00^c^ |
|  | *Enterobacteriaceae* | 0.00^e^ | 0.00^de^ | 0.00^e^ | 0.34^a^ | 0.01^de^ | 0.04^de^ | 0.08^c^ | 0.24^b^ |
| Proteobacteria/Moraxellaceae | *Acinetobacter* sp. | 0.45^c^ | 0.27^cd^ | 0.26^cd^ | 1.52^a^ | 0.06^d^ | 0.08^d^ | 0.46^c^ | 0.99^b^ |
|  | *Moraxella osloensis* | 0.03^b^ | 1.70^a^ | 0.01^b^ | 0.00^b^ | 0.00^b^ | 0.06^b^ | 0.00^b^ | 0.00^b^ |
| Proteobacteria/Pseudomonadaceae | *Pseudomonas* sp. | 0.18^f^ | 0.22^f^ | 0.02^f^ | 39.75^d^ | 43.09^c^ | 7.93^e^ | 53.87^a^ | 46.13^b^ |

*^a^* Within the same row, values sharing one or more superscript letters are not significantly (P > 0.05) different. Only OTUs with a relative abundance > 0.1% in at least one thesis are shown.

*^b^* Control, cheese made from whole milk and cream; RC, cheese made from semi-skimmed milk and reduced-fat cream; RCE1-2, cheese made from semi-skimmed milk and reduced-fat cream both added with E1 and E2; RCXE1, cheese made from semi-skimmed milk added with E1 and reduced-fat cream diluted with xanthan.
