# Supplementary material for: Use of Exopolysaccharide-Synthesizing Lactic Acid Bacteria and Fat Replacers for Manufacturing Reduced-Fat Burrata Cheese: Microbiological Aspects and Sensory Evaluation
Source: Microorganisms. 2020 Oct 21;8(10):1618. doi: 10.3390/microorganisms8101618 (PMC7588969; doi:10.3390/microorganisms8101618)
Supplement: Supplementary file 1 [file microorganisms-08-01618-s001.zip › Costantino et al._supplementary figures.pptx]

## Slide 1
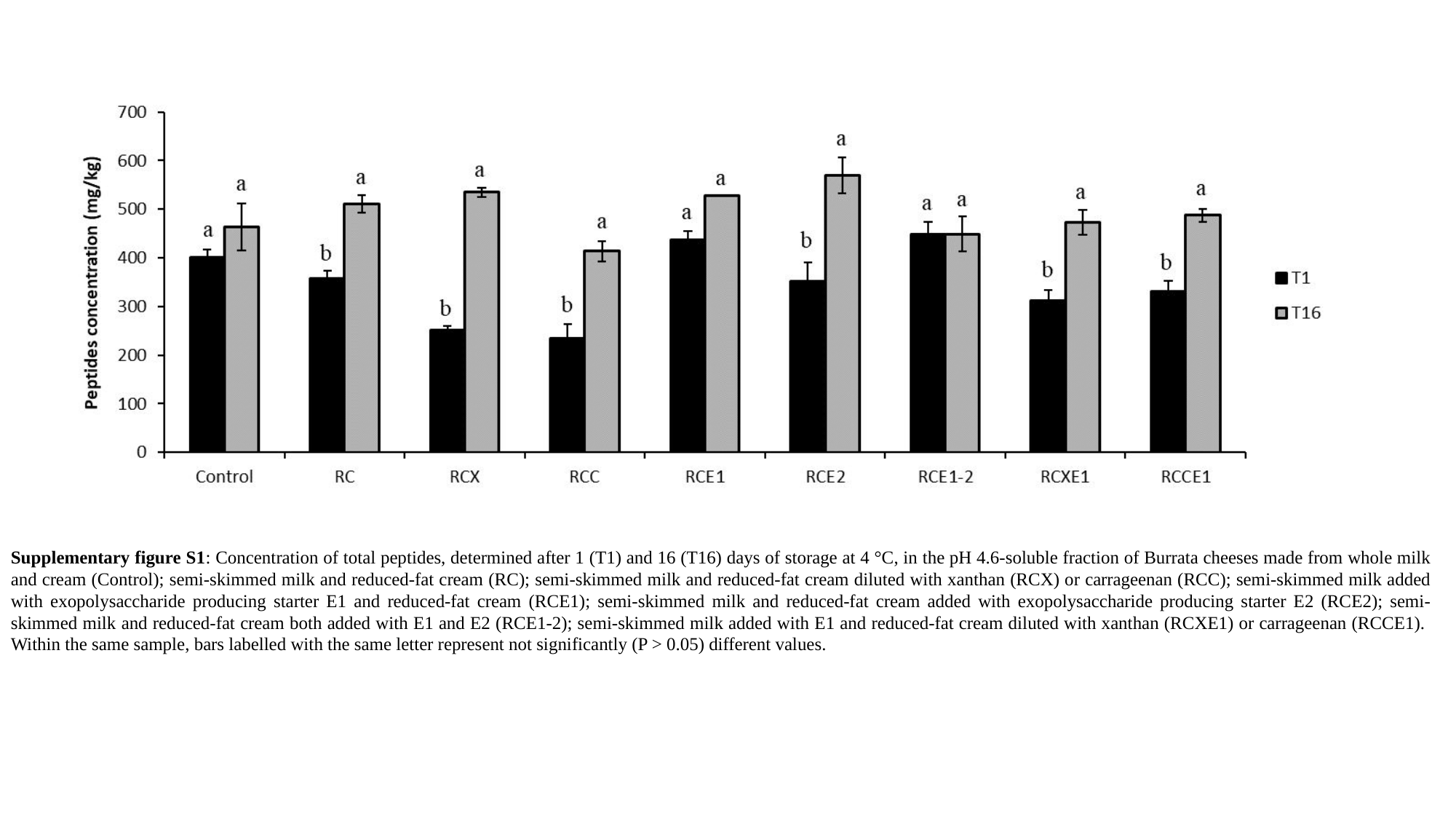

Supplementary figure S1: Concentration of total peptides, determined after 1 (T1) and 16 (T16) days of storage at 4 °C, in the pH 4.6-soluble fraction of Burrata cheeses made from whole milk and cream (Control); semi-skimmed milk and reduced-fat cream (RC); semi-skimmed milk and reduced-fat cream diluted with xanthan (RCX) or carrageenan (RCC); semi-skimmed milk added with exopolysaccharide producing starter E1 and reduced-fat cream (RCE1); semi-skimmed milk and reduced-fat cream added with exopolysaccharide producing starter E2 (RCE2); semi-skimmed milk and reduced-fat cream both added with E1 and E2 (RCE1-2); semi-skimmed milk added with E1 and reduced-fat cream diluted with xanthan (RCXE1) or carrageenan (RCCE1). Within the same sample, bars labelled with the same letter represent not significantly (P > 0.05) different values.

## Slide 2
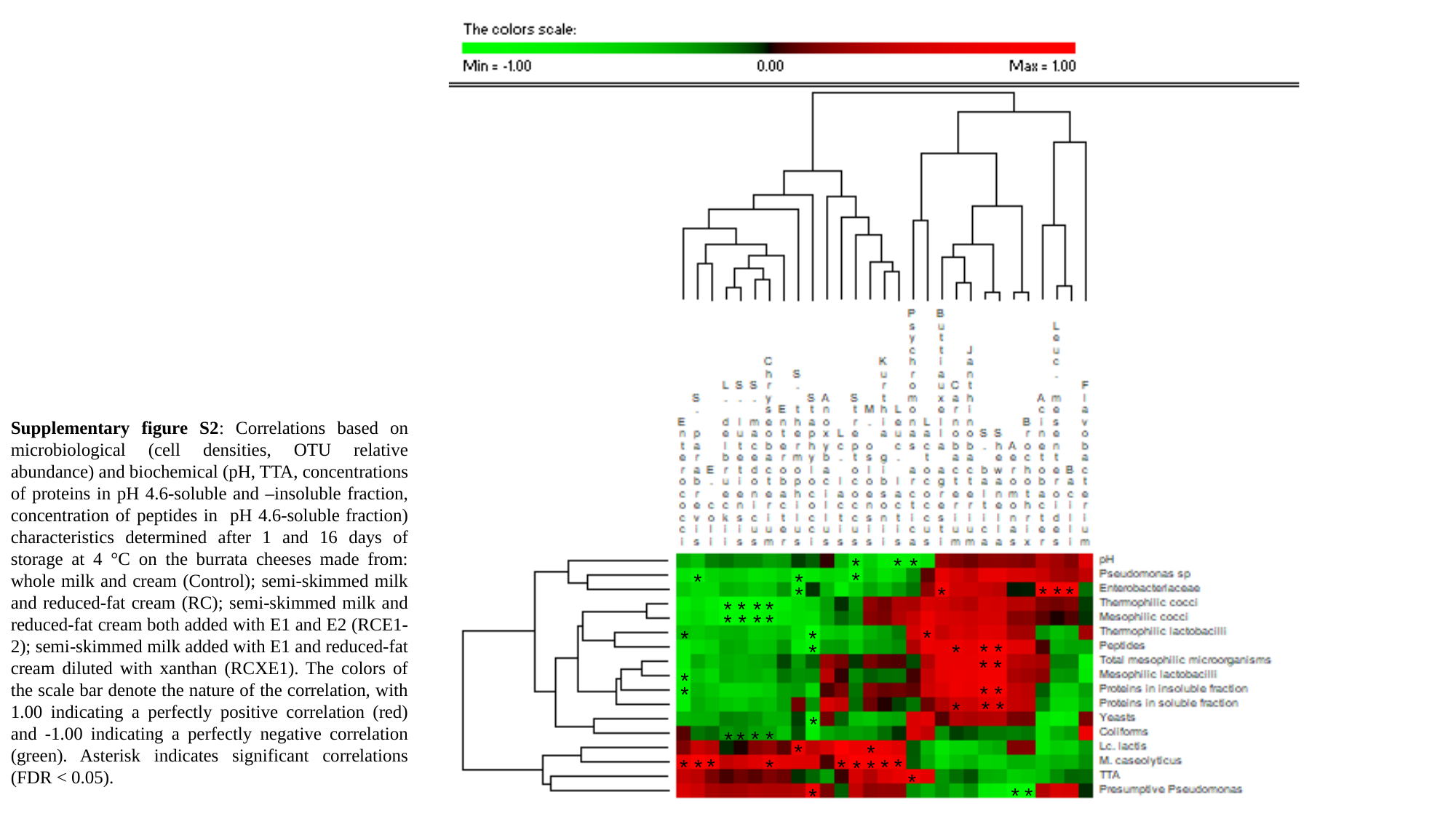

*
*
*
*
*
*
*
*
*
*
*
*
*
*
*
*
*
*
*
*
*
*
*
*
*
*
*
*
*
*
*
*
*
*
*
*
*
*
*
*
*
*
*
*
*
*
*
*
*
*
*
*
*
*
*
Supplementary figure S2: Correlations based on microbiological (cell densities, OTU relative abundance) and biochemical (pH, TTA, concentrations of proteins in pH 4.6-soluble and –insoluble fraction, concentration of peptides in pH 4.6-soluble fraction) characteristics determined after 1 and 16 days of storage at 4 °C on the burrata cheeses made from: whole milk and cream (Control); semi-skimmed milk and reduced-fat cream (RC); semi-skimmed milk and reduced-fat cream both added with E1 and E2 (RCE1-2); semi-skimmed milk added with E1 and reduced-fat cream diluted with xanthan (RCXE1). The colors of the scale bar denote the nature of the correlation, with 1.00 indicating a perfectly positive correlation (red) and -1.00 indicating a perfectly negative correlation (green). Asterisk indicates significant correlations (FDR < 0.05).
